# Factors for Differential Outcome Across Cancers in Clinical Molecule-Targeted Fluorescence Imaging

**DOI:** 10.2967/jnumed.121.263674

**Published:** 2022-11

**Authors:** Quan Zhou, Nynke S. van den Berg, Wenying Kang, Jacqueline Pei, Naoki Nishio, Stan van Keulen, Myrthe A. Engelen, Yu-Jin Lee, Marisa Hom, Johana C.M. Vega Leonel, Zachary Hart, Hannes Vogel, Romain Cayrol, Brock A. Martin, Mark Roesner, Glenn Shields, Natalie Lui, Melanie Hayden Gephart, Roan C. Raymundo, Grace Yi, Monica Granucci, Gerald A. Grant, Gordon Li, Eben L. Rosenthal

**Affiliations:** 1 Department of Neurosurgery, Stanford University School of Medicine, Stanford, California;; 2 Department of Otolaryngology, Stanford University School of Medicine, Stanford, California;; 3 Department of Otorhinolaryngology, Nagoya University Graduate School of Medicine, Nagoya, Japan;; 4 Department of Oral and Maxillofacial Surgery and Oral Pathology, Amsterdam UMC–location VUMC/Academic Centre for Dentistry Amsterdam, Amsterdam, The Netherlands;; 5 Department of Mechanical Engineering, Delft University of Technology, Delft, The Netherlands;; 6 Department of Pathology, Stanford University, Stanford, California;; 7 Stanford Health Care, Stanford University Medical Center, Stanford, California;; 8 Department of Cardiothoracic Surgery, Stanford University Medical Center, Stanford, California; and; 9 Cancer Clinical Trials Office, Stanford University School of Medicine, Stanford, California

**Keywords:** clinical fluorescence imaging, epidermal growth factor receptor, multicancer surgical imaging, physical and biologic factors, panitumumab-IRDye800

## Abstract

Clinical imaging performance using a fluorescent antibody was compared across 3 cancers to elucidate physical and biologic factors contributing to differential translation of epidermal growth factor receptor (EGFR) expression to macroscopic fluorescence in tumors. **Methods:** Thirty-one patients with high-grade glioma (HGG, *n* = 5), head-and-neck squamous cell carcinoma (HNSCC, *n* = 23), or lung adenocarcinoma (LAC, *n* = 3) were systemically infused with 50 mg of panitumumab-IRDye800 1–3 d before surgery. Intraoperative open-field fluorescent images of the surgical field were acquired, with imaging device settings and operating room lighting conditions being tested on tissue-mimicking phantoms. Fluorescence contrast and margin size were measured on resected specimen surfaces. Antibody distribution and EGFR immunoreactivity were characterized in macroscopic and microscopic histologic structures. The integrity of the blood–brain barrier was examined via tight junction protein (Claudin-5) expression with immunohistochemistry. Stepwise multivariate linear regression of biologic variables was performed to identify independent predictors of panitumumab-IRDye800 concentration in tissue. **Results:** Optimally acquired at the lowest gain for tumor detection with ambient light, intraoperative fluorescence imaging enhanced tissue-size dependent tumor contrast by 5.2-fold, 3.4-fold, and 1.4-fold in HGG, HNSCC, and LAC, respectively. Tissue surface fluorescence target-to-background ratio correlated with margin size and identified 78%–97% of at-risk resection margins ex vivo. In 4-μm-thick tissue sections, fluorescence detected tumor with 0.85–0.89 areas under the receiver-operating-characteristic curves. Preferential breakdown of blood–brain barrier in HGG improved tumor specificity of intratumoral antibody distribution relative to that of EGFR (96% vs. 80%) despite its reduced concentration (3.9 ng/mg of tissue) compared with HNSCC (8.1 ng/mg) and LAC (6.3 ng/mg). Cellular EGFR expression, tumor cell density, plasma antibody concentration, and delivery barrier were independently associated with local intratumoral panitumumab-IRDye800 concentration, with 0.62 goodness of fit of prediction. **Conclusion:** In multicancer clinical imaging of a receptor-ligand–based molecular probe, plasma antibody concentration, delivery barrier, and intratumoral EGFR expression driven by cellular biomarker expression and tumor cell density led to heterogeneous intratumoral antibody accumulation and spatial distribution whereas tumor size, resection margin, and intraoperative imaging settings substantially influenced macroscopic tumor contrast.

## Visual Abstract

**Figure:**
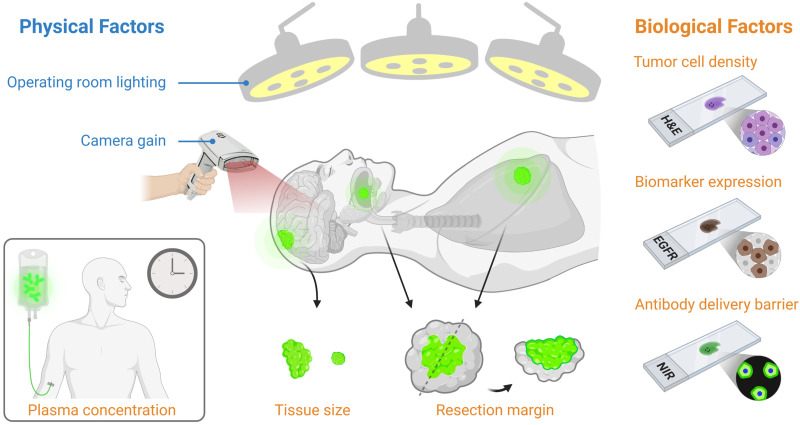

Intraoperative surgical imaging with tumor-specific fluorescent tracers offers additional tumor contrast for surgeons, who rely heavily on visual cues for resection decisions. In recent years, receptor-ligand–based imaging probes have achieved early successes in detecting cancers of the head and neck, brain, ovary, pancreas, kidney, prostate, and colon (*1**–**7*). Yet how biomarker expression translates to fluorescence and clinical imaging outcome remains unexamined. As more molecular imaging probes enter late-phase clinical trials, we compared the performance of a fluorescently labeled epidermal growth factor receptor (EGFR) antibody, panitumumab-IRDye800, in different tumor types to elucidate intrinsic and extrinsic parameters that influence tumor imaging and inform clinical decisions.

Our primary objective was to examine, in multiple cancers, physical and biologic factors that contributed to differential fluorescence imaging outcome in terms of intraoperative tumor contrast, pathologic margin assessment, and fluorescent antibody distribution. Various combinations of biomarker targets, molecular probes, imaging devices and analysis methods among imaging studies make collective interpretation of their findings challenging. For trials with dose escalation design, results between cohorts can be naturally reported within the same analysis framework as illustrated in breast cancer (*8*). However, no consensus exists yet to evaluate a molecular probe among multiple cancers. We therefore acquired and processed fluorescence images across malignancies with the same imaging and analysis pipeline to identify attributes that bridge the gap between molecular characteristics and imaging outcome in fluorescence-guided surgery.

## MATERIALS AND METHODS

### Participants

The open-label phase I/II clinical trials NCT03510208, NCT02415881, and NCT03582124 were conducted in adult patients undergoing surgical resection at Stanford Hospital for high-grade glioma (HGG, *n* = 5), head-and-neck squamous cell carcinoma (HNSCC, *n* = 25), and lung adenocarcinoma (LAC, *n* = 3), respectively. Between August 2017 and November 2019, 33 enrolled patients received a single dose of 50 mg of panitumumab-IRDye800 (produced following current good manufacturing practices by the Frederick National Laboratory) regardless of weight or sex 1–3 d before surgery. Adverse events were collected up to 30 d after infusion. Two HNSCC patients were excluded from final analysis as no cancer was found in their resected tissue. The maximum dimension of tumor size was determined by presurgical MRI or CT. Areas of viable tumor and of normal tissue were outlined by board-certified pathologists on representative histology staining of tissue sections. The Institutional Review Board approved this study, and all subjects gave written informed consent.

### Fluorescence Imaging

**FIGURE 1.**
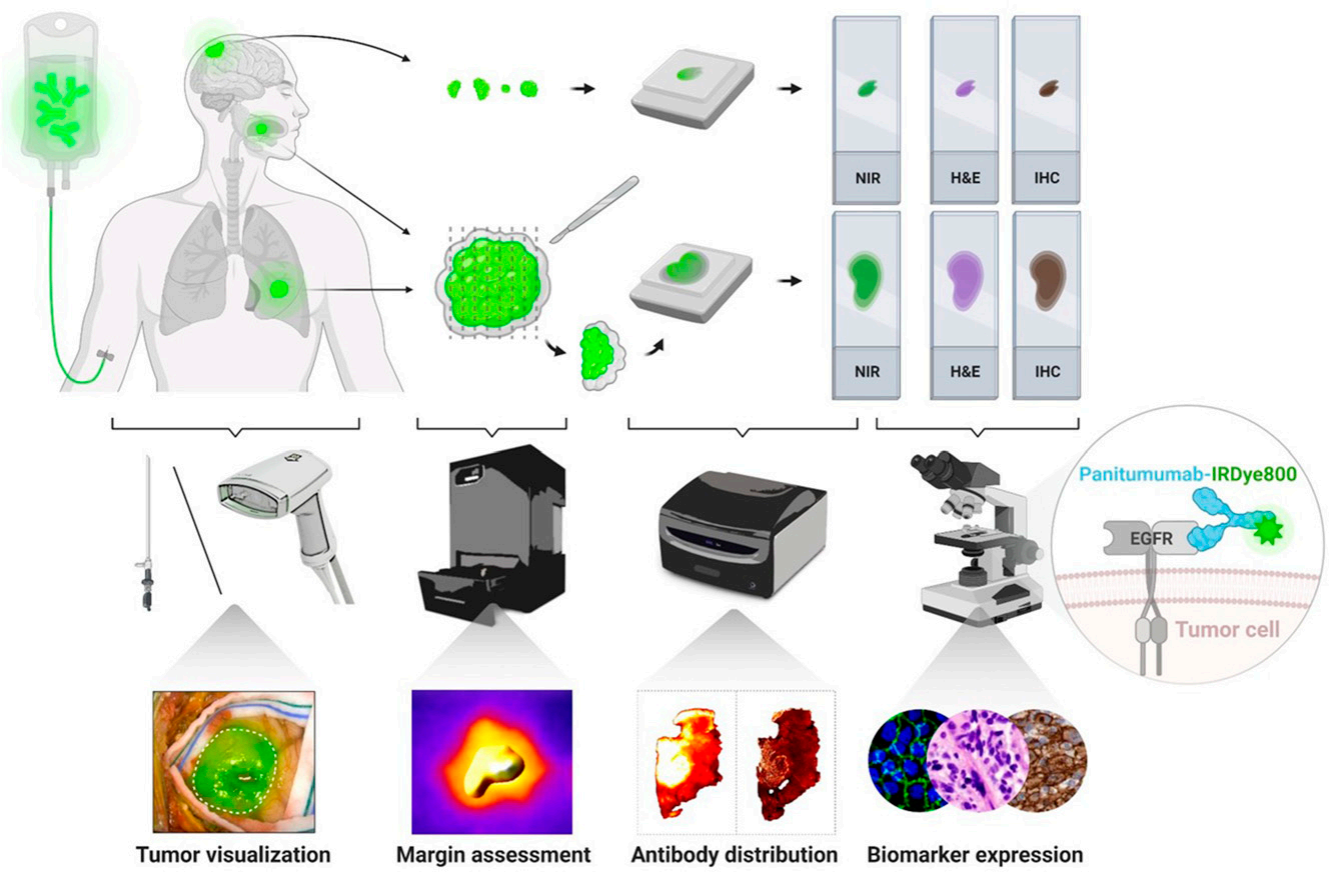
Tissue processing and imaging workflow. H&E = hematoxylin and eosin; IHC = immunohistochemistry.

As illustrated in the tissue processing and imaging workflow (Fig. 1), a laparoscope or a handheld camera attached to the SPY fluorescence imaging platform (Novadaq) detected intraoperative near-infrared (NIR) fluorescence of the tumor and wound bed. Intraoperative blood samples were collected to measure plasma panitumumab-IRDye800 concentration. Solid tumors were resected en bloc, whereas diffuse HGGs were removed in pieces. Ex vivo fresh tissue was imaged in the Pearl Trilogy Imaging System (LI-COR Biosciences) without ambient light. Solid tumors were fixed and sectioned into 5-mm-thick serial cross sections and paraffin-embedded in tissue blocks. Histologic and immunohistochemical stainings were performed on 4-μm-thick tissue sections. Fluorescence images of both tissue blocks and sections were acquired in an Odyssey CLx scanner (LI-COR). The distance from the tissue resection surface to the solid tumor margin was measured on histology sections.

### Fluorescence Quantification

Tumor contrast was measured by the ratio of average pixel intensities (ImageJ, version 1.53c (*9*)) from 5 circular regions of interest (diameter, 20 pixels, with *x-* and *y*-coordinates determined from randomly generated integer pairs) inside tumor and surrounding normal areas in intraoperative white-light and fluorescence images. Fluorescence histograms were plotted for the entire tumor and peritumoral normal areas. High-intensity peaks in the fluorescence map of resected tissue were isolated as previously described (*10*). Mean fluorescence intensity (MFI) was measured in Image Studio (LI-COR) as total fluorescence signal divided by the pixel number within regions of interest. MFI in normal tissue was measured in muscle or brain tissue with less than 20% tumor cells. Tumor-to-background ratio on fresh resected tissue surface denoted the ratio of MFIs in circular regions of interest (diameter, 2 mm) over tumor versus those over normal tissue. Tumor-to-background ratio of tissue sections was the ratio of MFI within outlined tumor versus uninvolved tissue. Fluorescence heterogeneity denoted the SD of fluorescence signal normalized by MFI. MFIs of anatomic structures (circular regions of interest; diameter, 200 μm) on tissue sections were measured.

### Tissue-Mimicking Phantoms

Serial dilutions of panitumumab-IRDye800 (0–10.0 g/mL) were respectively dissolved in 1% agarose (Life Technologies) and 1% intralipid (Sigma-Aldrich) at 45°C and poured into 200-μL cylindric molds. Solidified phantoms were imaged (SPY platform, gain: 2, 4, and 8) in the operating room under 3 lighting conditions (ambient lights: TL-D, 36 W, Philips; room lights: A19, 100 W, Osram; overhead lights: F528, 140 W, Stryker). The ratio of MFIs between panitumumab-IRDye800 and saline-containing phantoms measured imaging contrast. Phantom MFIs measured in Pearl and Odyssey were correlated. Panitumumab-IRDye800 concentrations and MFIs of 4-μm phantom sections were fitted by polynomial regression.

### Immunohistochemistry

EGFR (RM-2111-RQ [Thermo Fisher Scientific]; secondary, SM805[Agilent Technologies]) immunohistochemistry and hematoxylin counterstaining were performed after heat-mediated antigen retrieval with Dako Autostainer (Agilent) along positive and negative controls. Double immunohistochemical staining of Claudin-5 (1:500, 34–1,600; Thermo Fisher) and ETS-related gene (1:1,000, EPR3864; Abcam) was performed on HGG tissue to assess blood–brain barrier (BBB) integrity (*2*). Immunoreactivity was visualized with diaminobenzidine (for EGFR and Claudin-5) and magenta (for ETS-related gene) chromogens (Dako) and scanned in NanoZoomer 2.0-HT (Hamamatsu Photonics). The percentage of pixels with moderate to strong staining was quantified with ImageScope (Aperio Technologies) as previously described (*11*). EGFR-positive tumor cells within tumor outlines were counted with a MATLAB (MathWorks) algorithm.

### Statistical Analysis

Group statistics were expressed as mean ± SE unless specified otherwise. Patient characteristics were compared between cancer types using ANOVA and Pearson χ^2^ tests as appropriate. Paired *t* tests (2-tailed) were performed for group comparisons between tumor and normal tissues in each cancer type. One-way ANOVA was performed for group comparisons among trials. Whiskers and outliers of box plots were determined by the Tukey method. Receiver-operating-characteristic curves were plotted for distinguishing histologic tumor versus normal tissue using MFI and EGFR. Sensitivity, specificity, area under the receiver-operating-characteristic curve, and negative and positive predictive values were subsequently calculated using these definitions. MFI and EGFR cutoffs that resulted in the maximal sensitivity and specificity combined were reported. Biologic variables were included in a stepwise multivariate linear regression model to identify independent predictors of local panitumumab-IRDye800 concentration. To exclude the possibility of false-positive associations, multicollinearity of predictors was assessed using the variance inflation factor, and predictors with a variance inflation factor of more than 5 were removed from the final model. Significance was defined at *P* values of less than 0.05, 0.01, 0.001, and 0.0001.

## RESULTS

### Clinical Data

No significant difference was found between trials in demographic features, weight-adjusted tracer dose, plasma panitumumab-IRDye800 concentration, and imaging window (Table 1). Although tumor size was similar among trials (*P* = 0.35), resected tissue size varied significantly between diffuse HGGs removed in pieces and solid tumors resected en bloc (16% ± 4% vs. 184% ± 20% of the tumor size, *P* = 0.0002) (Supplemental Fig. 1; supplemental materials are available at http://jnm.snmjournals.org). No infusion reactions or dose-limiting toxicity events occurred (Supplemental Table 1).

**TABLE 1.** Patient Characteristics

Characteristic	HGG (*n* = 5)	HNSCC (*n* = 23)	LAC (*n* = 3)	Total (*n* = 31)	*P*
Age (y)	62 (42–72)	67 (44–82)	71 (67–71)	67 (42–82)	0.41*
Sex, male	2 (40%)	10 (43%)	1 (33%)	13 (42%)	0.94^†^
Race					0.31^†^
Asian	1 (20%)	2 (9%)	1 (33%)	4 (13%)	
White	4 (80%)	20 (87%)	2 (67%)	26 (84%)	
Unknown/not reported	0 (0%)	1 (4%)	0 (0%)	1 (3%)	
Tumor size (cm)	5.0 (3.5–6.1)	2.8 (1.0–9.0)	2.3 (1.9–3.5)	3.7 (1.0–9.0)	0.35*
Pan800 dose (mg/kg)	0.8 ± 0.3	0.8 ± 0.2	0.6 ± 0.3	0.7 ± 0.2	0.84*
Pan800 DOS plasma concentration (mg/L)	6.9 ± 3.5	5.5 ± 4.2	3.9 ± 3.7	5.5 ± 4.0	0.58*
Imaging window (d)	1.8 (0.6–2.9)	1.8 (0.8–3.8)	1.7 (0.9–1.8)	1.8 (0.6–3.8)	0.95*

* One-way AVOVA.

† Pearson χ^2^ test.

Pan800 = panitumumab-IRDye800; DOS = day of surgery.

Qualitative data are number and percentage; continuous data are median and range or mean ± SD.

### Intraoperative Tumor Visualization

**FIGURE 2.**
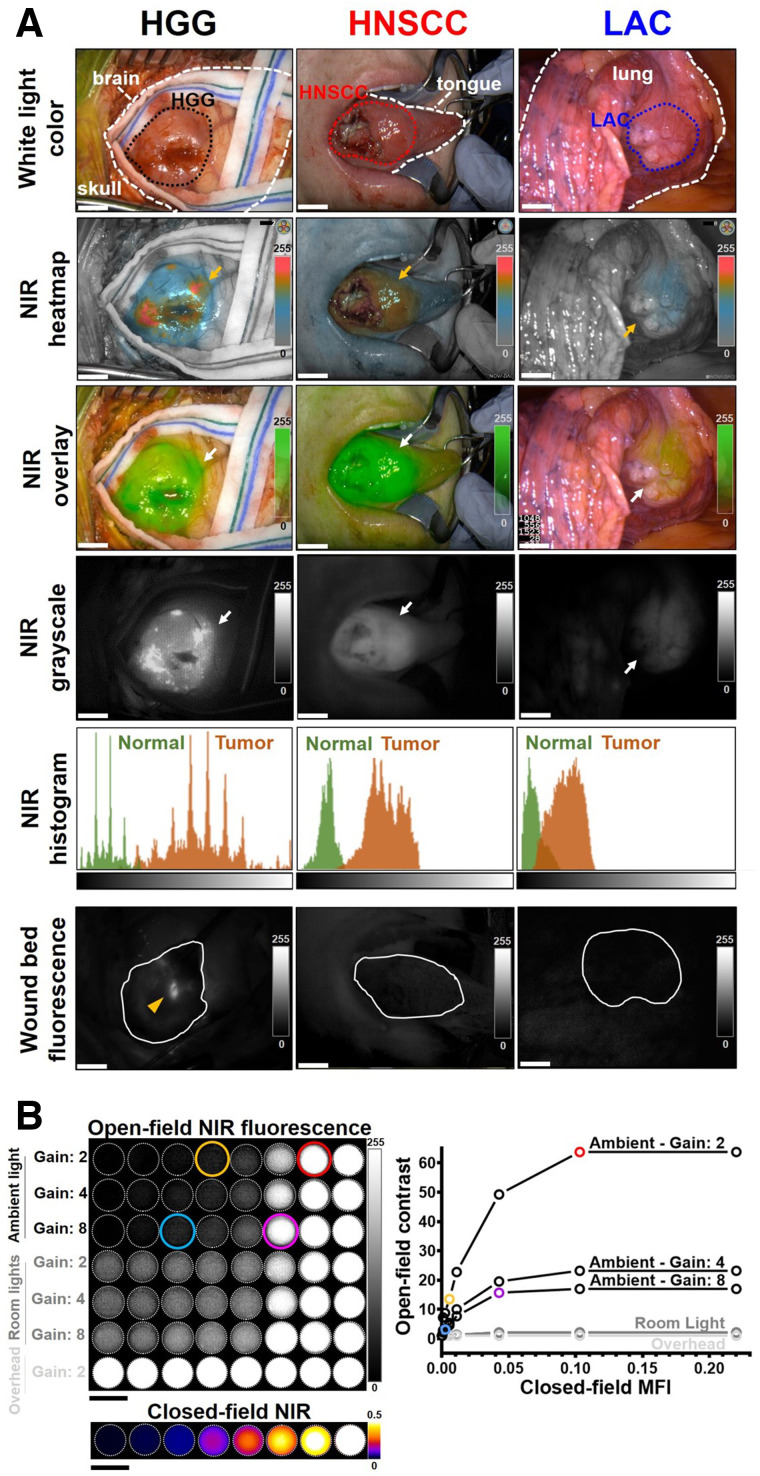
Intraoperative NIR fluorescence imaging enhanced tumor contrast in vivo. (A) Representative annotated (dashed lines) white-light photographs and fluorescence images of exposed tumors (dotted outlines) and wound beds (solid outlines) in surgical field. Arrows = positive NIR fluorescence signal; arrowhead = residual tumor; histogram (of NIR grayscale images) *x*-axis = pixel fluorescence intensity (range, 0–255); *y*-axis = pixel count (range, 0–5,000); scale bars = 1 cm. (B) NIR fluorescence images of tissue-mimicking phantoms containing serial dilutions of panitumumab-IRDye800 (0–10 μg/mL) acquired in either open-field imager under 3 lighting conditions with various gain settings or closed-field device. Scale bars = 1 cm.

Intraoperative fluorescence was diffuse in LAC compared with the strong signal in HGG and HNSCC that allowed distinct separation of disease tissue from normal areas based on histologic confirmation, with notable heterogeneity in HGG (Fig. 2A). Minimal fluorescence remained in the wound beds of HNSCC and LAC, whereas fluorescent residual HGG involving eloquent cortex located beyond contrast-enhancing margin was not removed in the resection cavity (Supplemental Fig. 2). NIR imaging enhanced tumor contrast relative to white-light illumination by 5.2-fold (*P* = 0.0006), 3.4-fold (*P* < 0.0001), and 1.4-fold (*P* = 0.03) for HGG, HNSCC, and LAC, respectively, and fluorescence contrast dropped below 1.0 in the wound beds (Supplemental Fig. 3). Ex vivo tissue fluorescence contrast correlated with resected tumor size (*P* = 0.007) (Supplemental Fig. 4).

The open-field fluorescence imaging had limited sensitivity and dynamic range over tissue-mimicking phantoms containing panitumumab-IRDye800, which were readily distinguished from each other without ambient light (Fig. 2B). Per workflow requirements, ambient lighting was always present in the operating room. Detection sensitivity was improved with higher gain (blue vs. yellow circles), at the cost of reduced saturation threshold (pink vs. red circles). Operating room lights gave false-positive signals, and images of control phantoms were saturated with overhead lights, indicating NIR interference from these light sources.

### Margin Assessment

**FIGURE 3.**
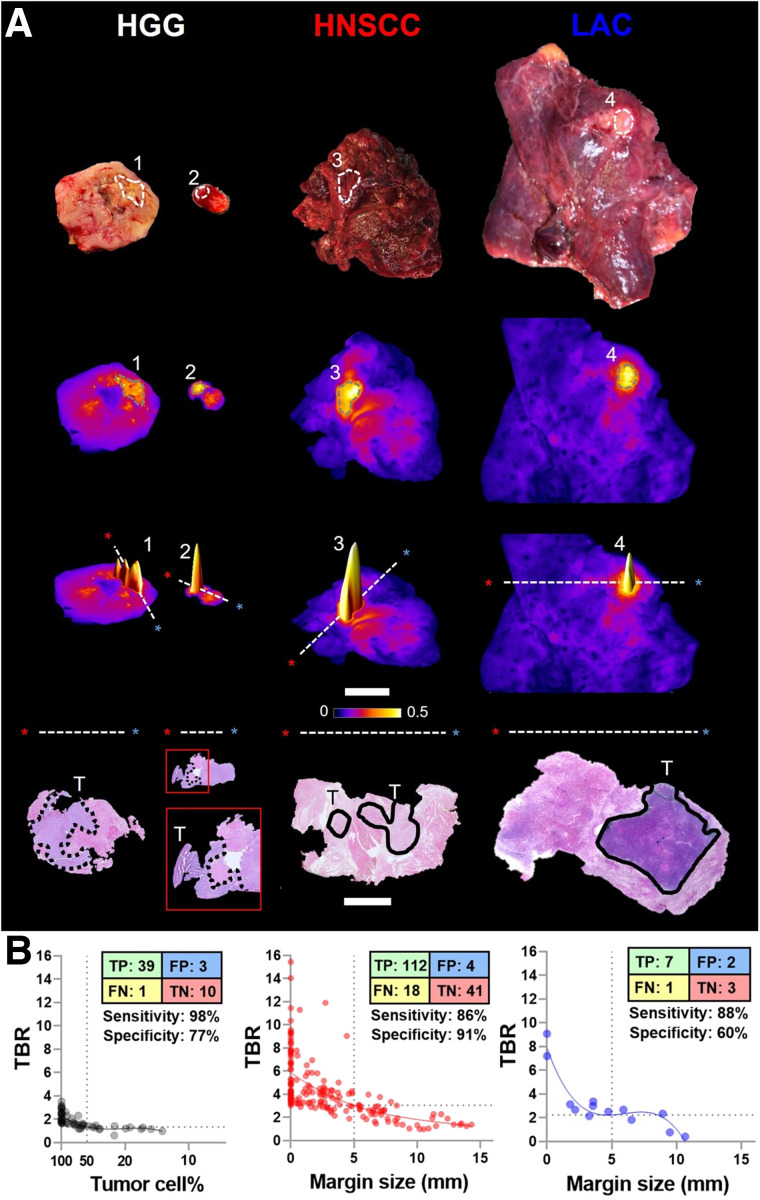
Macroscopic closed-field NIR imaging identified at-risk margins in resected tissue. (A) Representative intraoperative photographs and fluorescence images of resected tissue specimens. 1–4 = fluorescence intensity peaks; scale bar = 2 cm; dashed lines and asterisks (red and blue) = orientation in which histology (hematoxylin and eosin) slides with infiltrative (dotted outlines) and solid (solid outlines) tumors were sectioned; scale bar = 1 cm. (B) Fluorescence target-to-background ratio (TBR) correlated with tumor cell percentage (HGG) and margin size (HNSCC and LAC). FN = false-negative; FP = false-positive; T = tumors; TN = true-negative; TP = true-positive.

Fluorescence intensity peaks on fluorescence images of resected tumor specimens identified at-risk margin (Fig. 3A). The HGG cell density decreased beyond the infiltrating edge, and distances from tissue surface to tumor margin were inversely correlated with fluorescence contrast on the specimen surface (Fig. 3B). Positive and close margins can be captured above the target-to-background value at 5 mm on fitted regression curves with 97% and 78% detection rates for HNSCC and LAC, respectively, whereas 93% HGG infiltrative edges with at least 50% tumor cell density were detected.

### Intratumoral Fluorescent Antibody Distribution

**FIGURE 4.**
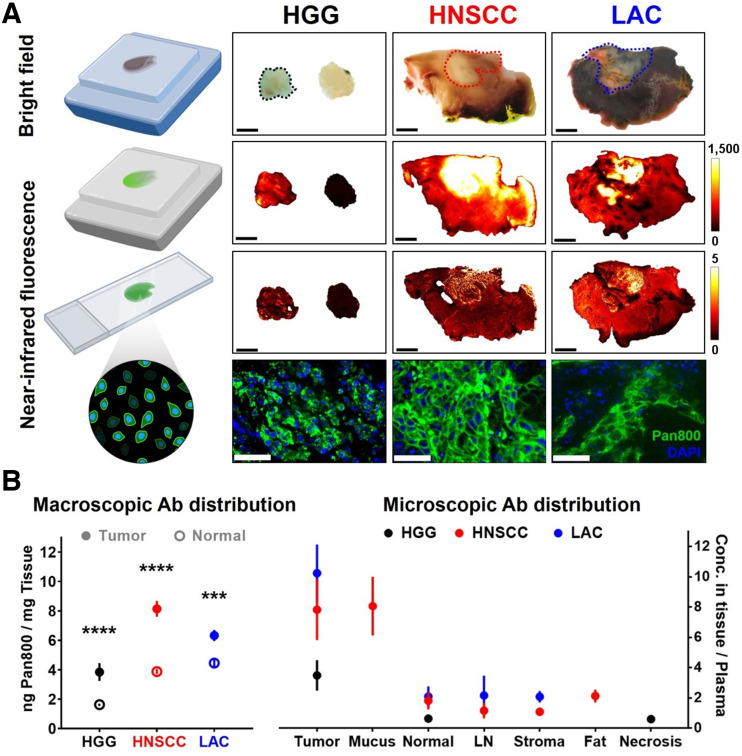
Intratumoral distribution and cellular delivery of fluorescent antibody. (A) Bright-field photographs (scale bars = 5 mm; dotted outlines = tumor) and fluorescence images (scale bars = 50 μm) of fixed tissue blocks and sections. (B) Macroscopic and microscopic distribution of panitumumab-IRDye800 in histologic tissue types. Ab = antibody. ***P < 0.001. ****P < 0.0001.

Microscopic NIR images of tissue blocks and sections exposing tumor interior confirmed intratumoral distribution and cellular delivery of panitumumab-IRDye800 (Fig. 4A). Fluorescence heterogeneity was more pronounced in HGG than in LAC (*P* = 0.02), with similar tumoral fluorescence contrast across cancers (Supplemental Fig. 5). Fluorescence in tissue sections can effectively distinguish tumor from normal tissue (area under the receiver-operating-characteristic curve: HNSCC > LAC > HGG = 0.85; Supplemental Fig. 6). Differences in tumor fluorescence converged from 244-fold to 21-fold (Supplemental Fig. 7) as variance in tissue thickness reduced from centimeters in fresh tissue to less than 1 μm in tissue sections, whereas their fluorescence intensity measurements by 2 closed-field devices were correlated (Supplemental Fig. 8).

Panitumumab-IRDye800 concentrations (inferred from fluorescence; Supplemental Fig. 9) were higher inside tumoral outlines than in healthy adjacent tissue of HGG (3.9 vs. 1.6 ng/mg, *P* < 0.0001), HNSCC (8.1 vs. 3.9 ng/mg, *P* < 0.0001), and LAC (6.3 vs. 4.5 ng/mg, *P* = 0.0006) (Fig. 4B). Further delineation into finer histologic structures revealed greater probe distribution in microscopic LAC tumor nodules relative to macroscopic tumoral area, indicating substantial presence of stroma with low antibody delivery inside LAC. Likely because of its EGFR expression, head-and-neck mucus exhibited distinctly high probe uptake among nontumoral areas, including normal tissue (muscle, lung, and brain), lymph node, stroma, fat, and necrosis.

### Biomarker Expression and Tumor Cell Density

**FIGURE 5.**
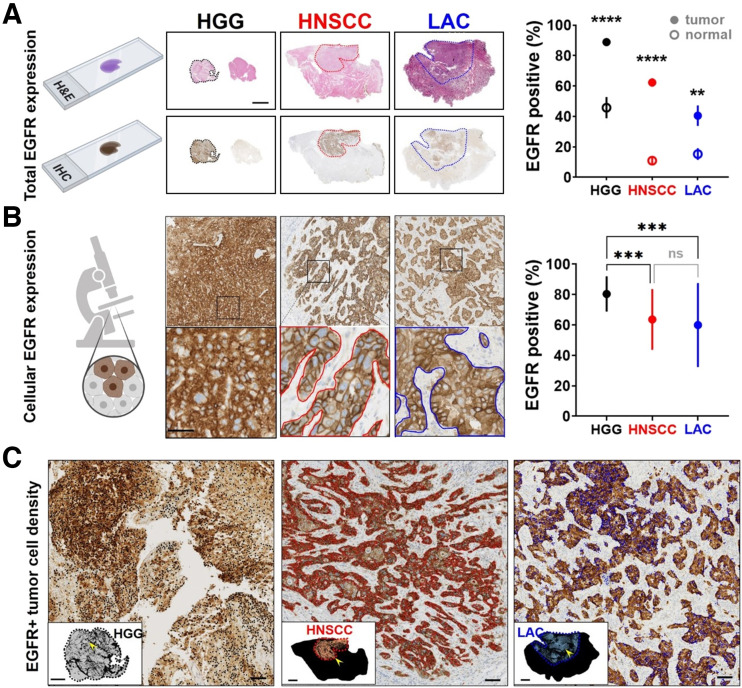
Heterogeneous EGFR expression in tumor. Tumor areas (scale bar = 5 mm; dotted outlines) on tissue sections of histologic (hematoxylin and eosin) and EGFR immunohistochemical stainings with total (A) and cellular (B) EGFR expression (scale bar = 50 μm; solid outlines). (C) EGFR-positive tumor cells (black = HGG; red = HNSCC; blue = LAC; scale bars = 200 μm) within tumor areas. Insets show distribution of EGFR-positive tumor cells on whole-tissue sections. Scale bar = 2 mm for HGG and 2 cm for HNSCC and LAC; arrowheads = location of high-magnification microscopic views. **P < 0.01. ***P < 0.001. ****P < 0.0001.

EGFR expression was heterogeneous (Fig. 5A), with greater fidelity for tumor presence in HNSCC and HGG than LAC (areas under the receiver-operating-characteristic curve, 0.96 and 0.94 vs. 0.82) (Supplemental Fig. 10). Nonspecific delivery to peritumoral EGFR-negative regions, however, was observed in head-and-neck as well as lung tissue (Figs. 4A and 5A). Higher total tumoral EGFR expression translated to greater panitumumab-IRDye800 concentration in tumors, with the notable exception of HGG (Figs. 4B and 5A), indicating a delivery barrier as confirmed by immunohistochemistry assessment of BBB integrity via the tight junction protein Claudin-5 (Supplemental Fig. 11). EGFR-positive HGG cells were diffuse, whereas focal clusters of HNSCC and LAC were dispersed among EGFR-negative stroma and fibroblast tissue (Fig. 5B).

The interplay of cellular expression, tumor cell density, and distribution pattern led to the scale-dependent nature of EGFR expression. Cellular EGFR level was particularly high in HGG (80% vs. 64% in HNSCC and 60% in LAC, *P* = 0.0005 and 0.001, respectively) but similar between HNSCC and LAC (*P* = 0.8). EGFR-positive tumor cells (Fig. 5C) were dense in HGG (3,000 ± 450 cells/mm^2^) and HNSCC (2,100 ± 180 cells/mm^2^) but sporadic in LAC (1,300 ± 100 cells/mm^2^), with fewer than 10 cells occupying over 50% of tumor areas (Supplemental Fig. 12). EGFR immunoreactivity thus varied with magnification powers and specific intratumoral locations examined (Supplemental Fig. 13).

### Multivariate Analysis

**FIGURE 6.**
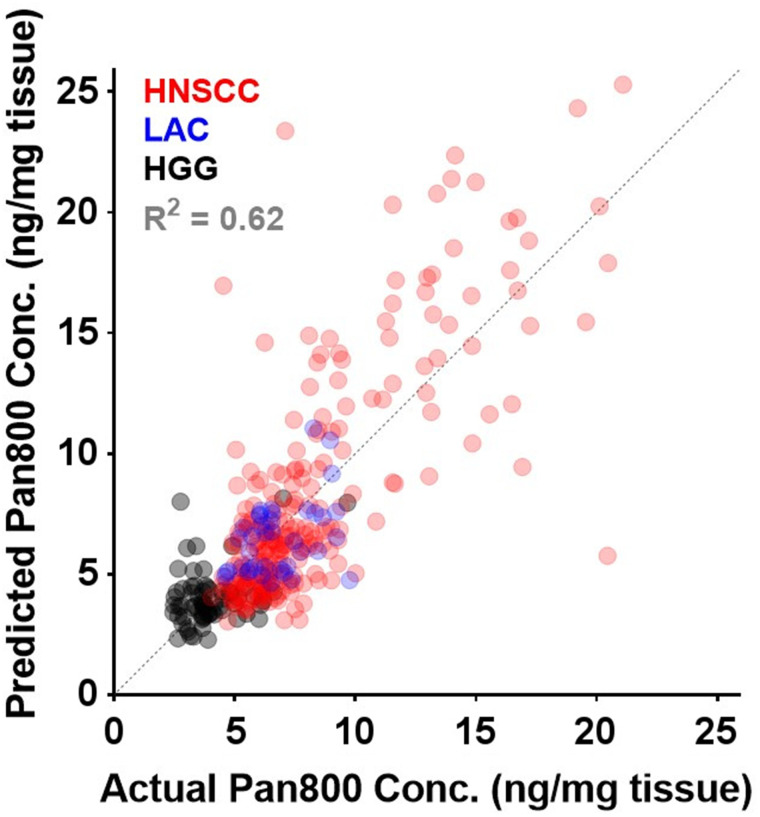
Goodness of fit for predicting local panitumumab-IRDye800 concentration from 4 biologic factors in multiple-regression model across 3 cancers.

In stepwise multiple linear regression analysis (insignificant independent variables removed one by one) controlled for other significant covariates such as tissue thickness, 4 biologic factors (Table 2, including tumor cell density (*P* = 0.015), cellular EGFR expression (*P* = 0.002), panitumumab-IRDye800 plasma concentration (*P* < 0.0001), and absence of delivery barrier (*P* < 0.0001), were independently associated (variance inflation factor = 1.08, 1.15, 1.16, and 1.08, respectively) with local intratumoral panitumumab-IRDye800 concentration (goodness of fit, 0.62; Fig. 6).

**TABLE 2.** Results of Multivariate Linear Regression Analysis

Variable	β	*P*
Tumor cell density	0.001447 (0.0002783, 0.002616)	0.0154
Cellular EGFR expression (%)	0.02561 (0.009501, 0.04171)	0.0019
Pan800 plasma concentration	4.498 (4.01, 4.986)	<0.0001
Delivery barrier (no)	2.119 (1.376, 2.861)	<0.0001

β = regression coefficient; Pan800 = panitumumab-IRDye800.

Data in parentheses are 95% CIs.

## DISCUSSION

In a receptor-ligand–based fluorescence imaging framework encompassing 3 cancers, we identified various factors that contributed to how biomarker expression translated to clinically relevant tumor imaging outcomes in terms of tumor contrast enhancement, at-risk margin detection, and fluorescent antibody distribution. Although cellular EGFR expression, tumor cell density, plasma antibody concentration, and delivery barrier may predict fluorescent antibody distribution in tissue, operating room lighting, imaging device settings, and tumor size and depth can substantially alter the intraoperative fluorescent tumor contrast at specific locations on the tissue surface of each particular patient. The interplay of these intrinsic and extrinsic attributes determined the differential translation of cellular biomarker expression to antibody uptake in tissue and ultimately the disparity in macroscopic fluorescent tumor contrast, with respective implications for projecting therapeutic antibody delivery and implementing surgical fluorescence imaging.

Physical imaging conditions and biologic tissue properties were isolated through the imaging-and-analysis pipeline. In particular, ex vivo closed-field fluorescence imaging eliminated ambient light and standardized acquisition settings that affected intraoperative open-field images. Similarly, in 4-μm-thick tumor cross sections, overlying normal tissue of resected whole-tissue specimens was removed and variable thickness of tumor tissue along the imaging path was equalized. In these optically transparent thin tissue sections, difference in light-scattering properties among tumor types was negligible to allow accurate quantification of fluorescence-based antibody distribution and antigen expression at microscopic resolution, revealing delivery barrier and tumor cell density as molecular and cellular underpinnings of their corresponding macroscopic characteristics.

To accommodate the wide range of interpatient fluorescence signal, minimal ambient light and the lowest imaging gain allowing tumor detection via fluorescence are recommended to maximize tumor-specific visual contrast in open-field intraoperative imaging, extending findings from previous phantom studies (*12**,**13*). Although only a few fluorescence peaks were sampled for pathologic assessment of head-and-neck cancer in previous studies (*10**,**14*), tissue surface fluorescent contrast was comprehensively characterized against margin distance in our study to identify tumor-to-background ratio cutoffs for detecting positive and close resection margins across 3 malignancies. In addition, rather than quantifying drug concentrations from tissue homogenate (*15*), we mapped antibody distribution to microscopic anatomic structures with high resolution (21 μm) and ultra sensitivity (2 pg of tissue) via fluorescence from intact tumor sections, preserving tissue integrity for downstream immunohistochemistry assays.

Our EGFR immunohistochemistry results revealed the scale-dependent and multifactorial nature of biomarker expression, reflecting its intrinsic intratumoral and interpatient heterogeneity across cancers. The Human Protein Atlas comparing EGFR expression among 20 cancer types reported moderate to strong immunoreactivity in 75% of patients with malignant glioma or head-and-neck cancer, followed by 64% of lung cancer patients (*16*). In the current study, total tumoral EGFR expression correlated with intratumoral antibody concentration, fluorescence, and tumor contrast, except for HGG, which had the highest total EGFR expression (89%, followed by HNSCC at 62% and LAC at 41%) yet received less than half the panitumumab-IRDye800 delivery observed in HNSCC (3.9 vs. 8.1 ng/mg), suggesting a delivery barrier, which was confirmed by immunohistochemical staining of tight junction protein.

The preferential intratumoral BBB breakdown by HGG promoted the tumor specificity of antibody distribution beyond that of the molecular target itself, despite introducing greater heterogeneity in fluorescence. Reduced tight junction protein expression around blood vessels indicated compromised BBB integrity in viable glioma tissue. Accordingly, specific cellular fluorescence confirmed panitumumab-IRDye800 delivery across leaky blood–tumor barrier, whereas intact BBB in normal brain tissue ensured minimal antibody delivery despite substantial EGFR expression, resulting in improved fluorescent tumor contrast consistent with prior preclinical (*17*) and clinical (*2**,**18**,**19*) evidence that even modest EGFR expression was sufficient for HGG detection with panitumumab-IRDye800.

In these early-phase clinical studies designed for safety and feasibility assessment, representation of certain populations was lacking and fluorescence was not used for intraoperative decision making per institutional review board protocols, whereas its tumor specificity warrants further efficacy studies in later-stage trials. Intraoperative wound bed imaging was more valuable for piecewise glioma resection (*2**,**20*) than solid tumor removed en bloc with a negative margin. Because of the dose-dependent nature of the panitumumab-IRDye800 half-life (14.5–24.8 h in the dose range of 0.06–1.5 mg/kg (*21*)), body weight and imaging window can influence the antibody plasma concentration and contribute to the variance in fluorescence. Therefore, antibody concentration in individual tissue types was normalized by corresponding day-of-surgery plasma concentration. Moreover, fluorescence signal was normalized by autofluorescence to mitigate variability in tissue section thickness (<2% (*22*)) and corrected for overestimation of antibody concentration (12.9%) due to shrinkage from tissue processing (11.4% (*23*)). Variable dye-to-protein ratios across production batches, photo bleaching, and metabolic rates can introduce noise and bias in methods using fluorescence as a surrogate for antibody presence. Thus, our fluorescence-based results are yet to be validated and calibrated by direct antibody quantification techniques such as mass spectrometry. Additional tissue properties, including collagen, stromal markers, and immune markers in the tumor microenvironment may also account for differential fluorescence intensity, which can be investigated in future studies.

## CONCLUSION

Cellular EGFR expression, tumor cell density, plasma antibody concentration, and delivery barrier determined the fluorescent antibody distribution in tissue, which differentially translated to macroscopic tumor contrast depending on tumor size, tumor depth, and intraoperative imaging conditions in HGG, HNSCC, and LAC patients infused with an NIR-labeled EGFR antibody. Potential clinical uses of molecule-targeted fluorescence imaging include intraoperative real-time tumor visualization, pathologic margin detection, and antibody distribution projection, with implications for oncologically sound resections, informed decision making on therapy, and regulatory approval of new imaging probes that have the potential to transform standard-of-care practice and patient care.

## DISCLOSURE

This work was supported in part by the Stanford Comprehensive Cancer Center, the Stanford University School of Medicine Medical Scholars Program, the Netherlands Organization for Scientific Research (Rubicon; 019.171LW.022), the National Institutes of Health and National Cancer Institute (R01CA190306), the National Institute on Deafness and Other Communication Disorders (T32DC015209), a scientific research grant from the Yokoyama Foundation for Clinical Pharmacology (YRY-1702), and an institutional equipment loan from LI-COR Biosciences Inc. Illustrations were created with BioRender. Eben Rosenthal acts as consultant for LI-COR Biosciences Inc. and has equipment loans from this company. No other potential conflict of interest relevant to this article was reported.

## Notes

KEY POINTS **QUESTION:** What physical imaging conditions and biologic tissue properties contributed to the differential translation of biomarker expression to clinical imaging outcomes in fluorescence-guided surgery? **PERTINENT FINDINGS:** In brain, head-and-neck, and lung cancer patients, cellular EGFR expression, tumor cell density, plasma antibody concentration, and delivery barrier predicted the heterogeneous intratumoral antibody distribution, whereas open-field imaging device settings, lighting conditions, and tumor size and depth substantially influenced the macroscopic fluorescence contrast. **IMPLICATIONS FOR PATIENT CARE:** By bridging the gap between molecular characteristics and imaging outcome, appropriate qualities of tumor biology and acquisition settings for optimal clinical implementation of targeted fluorescence imaging may facilitate assessment and adoption of emerging theranostic imaging probes.
